# Effect of Adherend Thickness on Near-Field Ultrasonic Welding of Single-Lap CF/LMPAEK Thermoplastic Composite Joints

**DOI:** 10.3390/ma16216968

**Published:** 2023-10-30

**Authors:** Natalia Sofia Guevara-Sotelo, Irene Fernandez Villegas

**Affiliations:** Aerospace Structures and Materials Department, Faculty of Aerospace Engineering, Delft University of Technology, 2629 HS Delft, The Netherlands; n.s.guevarasotelo@tudelft.nl

**Keywords:** fusion bonding, ultrasonic welding, thermoplastic composites, adherend thickness, hammering effect

## Abstract

Ultrasonic welding is a fast and promising joining technique for thermoplastic composite parts. Understanding how changing the part thickness affects the process is crucial to its future upscaling and industrialization. This article presents an initial insight into the effect of the adherend’s thickness on the near-field ultrasonic welding of CF/LMPAEK thermoplastic composites. Different thicknesses of the top and bottom adherend were welded and analyzed using the output data of the welding equipment, temperature measurements, and other visual characterization techniques. Increasing the thickness of both the top and the bottom adherends showed to increase the power consumed during welding. An overshoot in the power needed at the onset of the welding process for increased thickness of the top adherend precluded welding beyond a threshold thickness of 4.72 mm. In the case of the thicker top adherends, there was also melting of the energy director and early fiber squeeze-out within the top adherend as a result of increased bulk heating. Increased bulk heating was hypothesized to be caused by increased hammering, as indicated by the amplitude readings for thicker adherends. Welding with a higher force, which is known to reduce hammering, corroborated this hypothesis as fiber squeeze-out within the top adherend was not observed. It is believed that hammering contributes to heating by causing an oscillatory impact excitation that is close to the natural frequencies of the system, which would result in amplification of the cyclic strain and subsequent increase in the viscoelastic heating in the adherend.

## 1. Introduction

**Figure 1:**
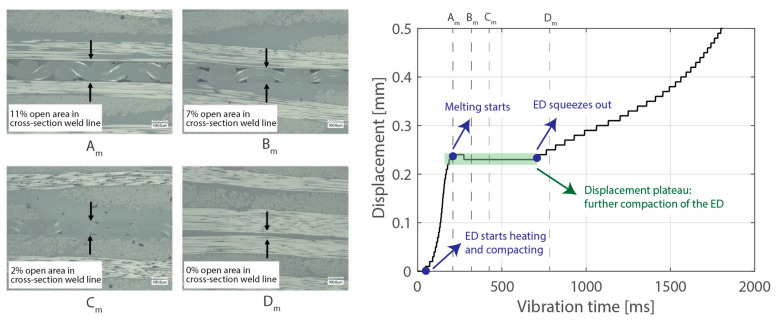
Cross-section micrographs showing the different steps a discontinuous energy director goes through under the ultrasonic vibrations, i.e., heating and further compaction followed by melting and squeeze-out (**left**). The same events can be identified in the downward displacement of the sonotrode (**right**). Adapted from [4].

Ultrasonic welding is a very interesting welding technique for thermoplastic composite assemblies mainly due to its ultra-fast heating rates and its ease of automation. Ultrasonic welding of thermoplastic composites is based on the application of high-frequency, typically 20 kHz, and low-amplitude mechanical vibrations perpendicular to the welding interface [1,2,3]. A sonotrode connected to a piezoelectric converter through a booster and to a press is used to transmit the vibrations into the material as well as to apply a certain static welding pressure throughout the welding process. Heat is generated through a combination of surface and viscoelastic friction. An energy director, in the form of resin-rich protrusions, a simple resin film, or a discontinuous resin film [4], is placed at the welding interface to ensure preferential heat generation at that location. As the vibrations are introduced in the welding stack (i.e., adherends and energy director), the energy director heats up, melts, and is (partially) squeezed out. Figure 1 shows such a succession of events for a discontinuous energy director. As a result, wetting of the adherends by the energy director occurs, which is a necessary condition for molecular inter-diffusion to ensue. As the final step, also known as the consolidation stage, the vibrations are stopped, and the weld is allowed to cool down under pressure. Ultrasonic welding is by nature a spot welding technique which, when applied sequentially, allows to create multi-spot welded overlaps [5,6]. When introducing a continuous relative movement between the welding and the parts to be joined (i.e., adherends), continuous ultrasonically welded overlaps can also be obtained [4,7,8].

In the last decade, there has been a renewed interest in the ultrasonic welding process, as indicated by the publication of numerous scientific articles on different aspects such as the impact of the morphology of the energy director [9,10], of the process parameters [11,12], and even of misaligned adherends [13,14] in the welding process. Interestingly, most of the research results reported in the open literature about ultrasonic welding of thermoplastic composites do not consider the thickness of the adherends as a variable. However, knowledge of the effect of the adherend thickness on the process and, especially, on its limits is of utmost importance for the selection of future applications for this welding technology. In the current state of the art, the thickness of the adherends is typically around 2 mm. In a previous paper by Fernandez Villegas on ultrasonic welding of carbon-fiber-reinforced polyetherimide composites [12], the author, however, doubled the thickness of the adherends (from 1.92 to 3.84 mm) as a side study to check the validity of some of the results obtained with 1.96 mm thick adherends concerning the use of the downward displacement of the sonotrode to control the weld quality. The results showed that the displacement value resulting in high-quality welds was the same in both cases; however, the overall power and energy consumed in the process were higher in the case of the thicker adherends. 

Contrarily, the topic of adherend thickness, or, more precisely, the distance between the tip of the sonotrode and the welding interface (*L*), has been discussed in greater measure in relation to ultrasonic welding of unreinforced thermoplastics. Based on that, two “types” of welding processes, near-field and far-field welding, have been defined based on whether *L* is lower or higher than a certain threshold value, respectively. Said threshold is considered to be 6 mm for usual thermoplastics, with wavelengths (*λ*) between 60 and 130 mm at 20 kHz [15,16]. During near-field welding, the amplitude of the vibrations reaching the welding interface can be considered similar to the amplitude of the vibrations exerted by the sonotrode. Contrarily, in far-field welding, the amplitude of the vibrations at the welding interface depends on wave propagation through the material, which is affected by the ratio between *L* and *λ* as well as by wave attenuation [1,17]. Consequently, far-field welding introduces additional requirements for the welding process to be successful (i.e., to produce similar results to near-field welding). This is especially the case when it comes to far-field welding of semi-crystalline plastics, which have higher energy requirements than amorphous plastics to be welded [1,17]. In particular, successful far-field welding of semi-crystalline thermoplastics requires the top adherend to be designed so that *L* is an integer multiple of 0.5*λ*. This ensures a displacement antinode and, hence, maximum heat generation at the energy director rather than at the interface between the sonotrode and the top adherend [1,17]. Given the typical *λ* values mentioned above (between 60 and 130 mm), this size requirement can be practical in, for instance, butt-welding of cylindrical hollow parts [17] but not so practical in, for instance, overlap-welding of thermoplastic composite laminates (assuming similar wavelengths in reinforced thermoplastics). 

This paper aims to provide initial insight into the effect of the adherend thickness on near-field ultrasonic welding of thermoplastic composites in a single-lap configuration. The material of choice for this research is carbon-fiber-reinforced low-melting polyaryletherketone (CF/LMPAEK), a semi-crystalline composite material of interest for high-performance, e.g., aerospace, applications. The research focuses on near-field ultrasonic welding given the typically thin-walled nature of high-performance thermoplastic composite structures. Based on previous experiments [12], it is expected that increasing the thickness of the adherends will, at the very least, increase the power consumed during the process, which might limit their applicability before the far-field threshold is reached. To look into this matter, the impacts of varying the thickness of the top adhered and of the bottom adherend were separately investigated. The regular output of the ultrasonic welding machine in terms of consumed power, downward displacement of the sonotrode, and amplitude was used as a first approach to observe changes in the welding process. Temperature measurements at the welding interface and within the adherends as well as high-speed camera recordings during the welding process were used to gain more insight into said changes.

## 2. Materials and Methods

### 2.1. Materials 

The composite material used for the adherends was TC1225 CF/LMPAEK provided by Toray Advanced Composites (The Netherlands). The material featured a five-harness satin T300JB woven carbon fiber reinforcement with 277 g/m^2^ fiber areal weight, 42% resin volume content, and nominal 0.31 mm consolidated ply thickness. The LMPAEK resin had a glass transition temperature of 147 °C and a melting temperature of 305 °C. Further, 580 mm x 580 mm composite laminates were manufactured by consolidating stacks of powder-impregnated composite layers in a hot platen Joos press (Pfalzgrafenweiler, Germany) at 365 °C and 10 bar for 30 min. Heating and cooling rates were set at 7 °C/min. For the thicker adherends, temperatures were checked during the consolidation cycle by placing K-type thermocouples (GG220-2K-0 provided by Tempco B.V., Bodegraven, The Netherlands) at the edges of the laminate (middle plane). The quality of the composite laminates was evaluated by C-scan (Olympus EPOCG 650, Hoofddorp, The Netherlands). Finally, the laminates were cut into single-lap shear adherends measuring 25.4 mm × 101.6 mm using a water-cooled Proth grinding machine (Taiwan). A 0.5 mm thick discontinuous LMPAEK film provided by Victrex (Middlesbrough, UK) was used for the energy directors. These discontinuous films present open areas. The energy directors were cut into rectangles slightly larger than the welding overlap (25.4 mm × 12.7 mm). Before welding, both the adherends and the energy directors were cleaned with a degreasing agent Hysol QD (PT Technologies Europe, Watergrasshill, Ireland).

Assuming the threshold between near-field and far-field welding to be at *L* = 6 mm (with *L* being the distance between the tip of the sonotrode and the welding interface, i.e., approximately the thickness of the top adherend), the adherend thicknesses considered in this study ranged from 1.17 mm (for a 4-ply laminate) to 5.79 mm (for a 20-ply laminate). Table 1 shows all the different thicknesses as well as laminate architectures used. The above-mentioned assumption was based on the fact that the wavelength of sound for the CF/LMPAEK material used in this study was measured to be 146 mm at 20 kHz (time of flight measurements on pulse-echo ultrasonic inspection, Olympus Omniscan MX with phased array technology). This is not very different from typical values provided in the literature for which the threshold between near field and far field is considered to be 6 mm [15,16]. 

### 2.2. Welding

A VE20 Slimline dialog 6200 ultrasonic welder from Herrmann Ultrasonics (Karlsbad, Germany) with a frequency of 20 kHz and 6.2 kW maximum power was used in this study. The welder was equipped with a rectangular sonotrode with a contact area of 15 mm × 30 mm and a gain of 1:1.7, and a booster with a gain of 1:2. With this setup, the welder can deliver a maximum peak-to-peak amplitude of 86.2 μm and a force between 130 and 2500 N. As the generic output of the ultrasonic welding process, the welder provides information about the power consumed during the process, and the actual amplitude and vertical displacement of the sonotrode. 

For the experiments performed in this study, the welding amplitude was set to 80 μm and the welding force to 500 N. These were chosen as a starting point based on typical force and amplitude values used in previous studies on ultrasonic welding of thermoplastic composites. Such values are not based on previous optimizations as the goal of this study was to gain an understanding of the effect of changing the thickness, and not to optimize the joint strength or other parameters. The duration of the vibration was either directly controlled (time control) or indirectly controlled through the downward displacement of the sonotrode. For the latter, the target displacement was set to 0.5 mm, i.e., the thickness of the energy director. This is further referred to as a “full weld”. It is known that such a long target displacement is beyond the point where high-quality welds are obtained [4,8], but, since this research did not aim at weld optimization, the long target displacement was chosen to provide a wide view of the welding process. The sonotrode was removed immediately after the vibration, meaning that there was no consolidation phase present in this study to avoid further material squeeze-out due to the applied pressure.

**Figure 2:**
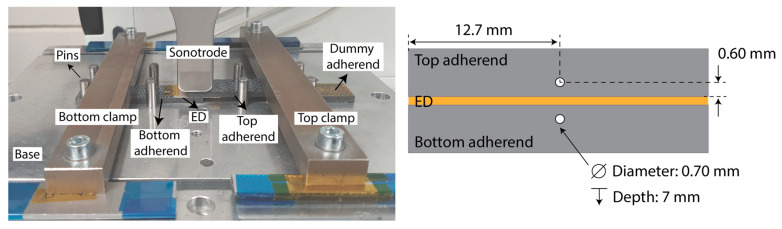
(**Left**): welding setup. (**Right**): schematic indicating thermocouple positioning.

The clamping jig used is shown in Figure 2. It consists of a base, bar clamps, and pins for positioning the adherends and it is designed to weld single-lap shear specimens in which the adherends have the dimensions 25.4 mm × 101.6 mm and the overlap is 25.4 mm × 12.7 mm. To ensure parallelism between the bottom and top adherends, a dummy adherend (with a thickness equal to that of the bottom adherend) and an energy director were placed below the top adherend. A summary of the experiments conducted in this study is presented in Table 2 (changing the top adherend’s thickness) and Table 3 (changing the bottom adherend’s thickness). It is important to note that the thickness of the bottom adherend was kept constant (1.83 mm) when changing the top adherend’s thickness and vice versa. The control parameter time was used only to stop the weld at a certain position in the displacement curve and it is not based on any previous optimization. Note that, for the case of repetitions with thermocouples, in some cases, the thermocouples failed, and the readings are therefore not presented in the results. 

### 2.3. Analysis

The power consumed, the downward displacement of the sonotrode, and the actual amplitude of vibration during the vibration phase of the welding process were directly obtained from the ultrasonic welder. 

Temperatures were measured at the welding interface and within the adherends using K-type thermocouples (GG220-2K-0, Tempco B.V., Bodegraven, The Netherlands) with a wire diameter of 0.10 mm and encapsulated diameter of 0.70 mm. For the temperature measurements within the adherends, holes were drilled in the adherends according to the schematic in Figure 2. The distance between the thermocouple hole and the interface (0.60 mm) was limited by the thickness of the thinner adherend (1.17 mm), and it was kept constant for all adherends to measure the temperature at the same position from the interface. For the temperature measurements at the welding interface, the measuring tip of the thermocouple was placed in the center of the overlap, on top of the bottom adherend and under the energy director. The thermocouples were connected to an analogue amplifier and sampled at 1 kHz. The temperature measurements were performed with three thermocouples (bottom adherend, interface, and top adherend). All thermocouples were placed simultaneously, which can affect the response of the welding process. However, because this added effect of placing thermocouples is present in all experiments when comparing different temperature evolutions for all thicknesses, it was not considered worrisome for this research. The raw thermocouple data were filtered in Matlab using a moving average filter with a 20-point window.

A FASTCAM NOVA S Series (Photron, Reutlingen, Germany) high-speed camera was used to record the welding process in slow motion using a rate of 1000 frames per second and a shutter speed of 1 μs. The camera was mounted on a tripod and positioned in such a way that the focus was on the middle of the welding stack. One extra lamp was required to achieve the desired lighting for the provided recording parameters.

As was mentioned earlier in the Introduction, the effects of changing the thickness of the top adhered and the bottom adherend were studied separately. Therefore, the welded joints will have a significant thickness mismatch between the top and bottom adherends. Because of this, it was decided not to perform any lap shear testing. Testing joints with different thicknesses and plainly comparing these values could be misleading because new variables, such as the bending stiffness which varies with thickness, are now involved.

## 3. Results

Only results are presented in this section. The analysis of the data, the trends, and further explanations are presented in the Discussion.

### 3.1. Thickness of the Top Adherend

**Figure 3:**
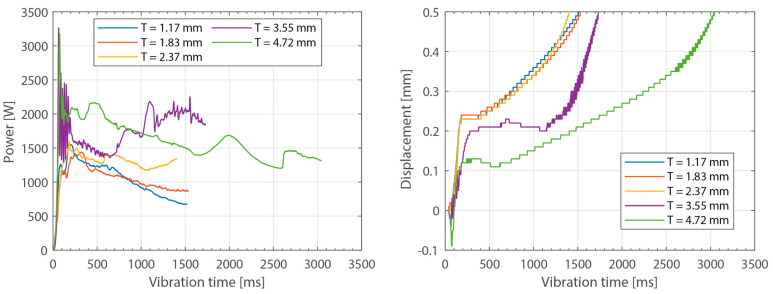
Consumed power (**left**) and downward displacement (**right**) curves for top adherend with varying thickness and discontinuous energy directors. One representative curve per thickness value. The welding parameters were 500 N force and 80 µm vibration amplitude. The bottom adherend’s thickness was 1.83 mm.

**Figure 4:**
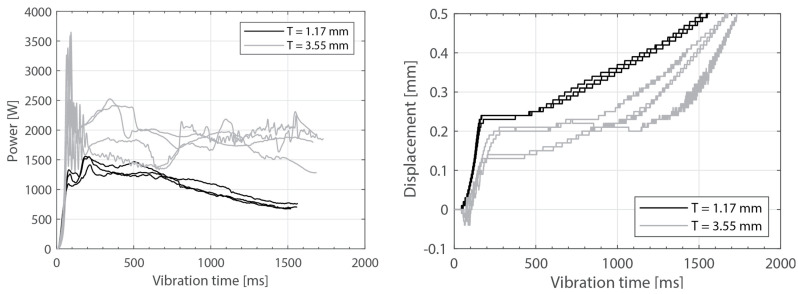
Consumed power (**left**) and downward displacement (**right**), three repetitions for two top adherend thickness values (1.17 mm and 3.55 mm). The welding parameters were 500 N force and 80 µm vibration amplitude. The bottom adherend’s thickness was 1.83 mm.

**Figure 5:**
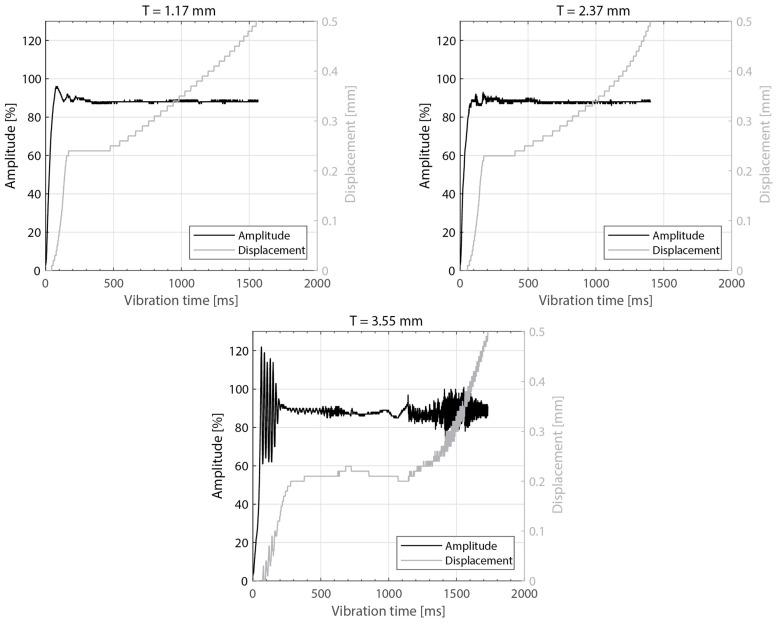
Amplitude and downward displacement curves for different thicknesses of the top adherend: 1.17 mm (**top-left**), 2.37 mm (**top-right**), and 3.55 mm (**bottom**). The welding parameters were 500 N force and 80 µm vibration amplitude. The bottom adherend’s thickness was 1.83 mm. The amplitude is provided as a percentage of the maximum amplitude the equipment can deliver.

**Figure 6:**
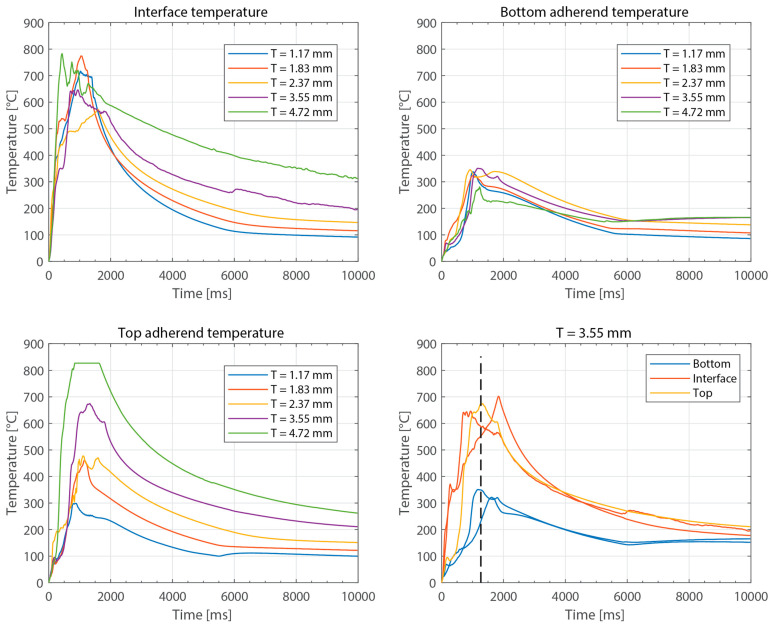
Representative temperature curves for different thicknesses of the top adherend: interface temperature (**top left**), bottom adherend temperature (**top right**), top adherend temperature (**bottom left**). (**Bottom right**): two repetitions for a top adherend thickness of 3.55 mm. For the temperature in the top adherend, only one thermocouple survived. The dashed line represents an estimate of the end of the vibrations. The welding parameters were 500 N force and 80 µm vibration amplitude. The bottom adherend’s thickness was 1.83 mm.

**Figure 7:**
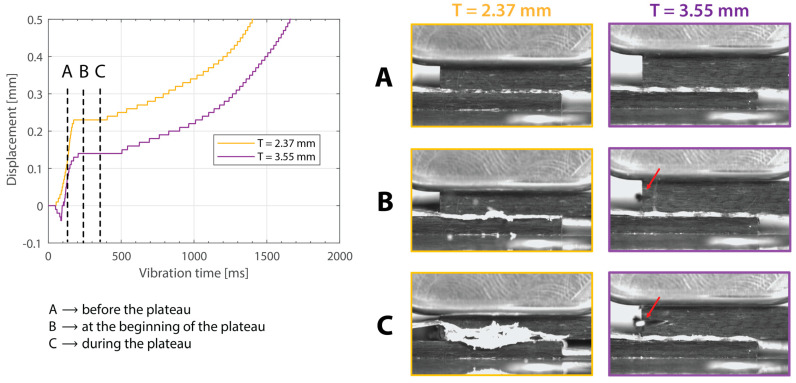
High-speed camera snapshots at different times in the welding process ((**A**) before the displacement plateau, (**B**) at the beginning of the displacement plateau, (**C**) during the displacement plateau) for two thicknesses of the top adherend (2.37 mm and 3.55 mm). The red arrows indicate fiber squeeze-out. The welding parameters were 500 N force and 80 µm vibration amplitude. The bottom adherend’s thickness was 1.83 mm.

Figure 3 shows the power consumed by the ultrasonic welder and the vertical downward displacement undergone by the sonotrode for five different thicknesses of the top adherend (ranging from 1.17 mm to 4.72 mm) and a fixed thickness of the bottom adherend (1.83 mm). One representative example is shown per thickness value. It should be noted that, due to a persistent error in the ultrasonic welder (maximum amplitude value exceeded), it was not possible to weld the configuration with a 5.79 mm thick top adherend, as originally intended. Figure 4 illustrates the repeatability of the results for two of those five thicknesses (1.17 mm and 3.55 mm). Figure 5 shows the amplitude as measured by the ultrasonic welder for three thicknesses of the top adherend with the corresponding sonotrode displacement curve superimposed for reference. Figure 6 shows temperature measurements at the welding interface within the bottom adherend and the top adherend for five different thicknesses and two repetitions for a top adherend thickness of 3.55 mm. Figure 7 gathers a selection of snapshots from the high-speed camera at three different stages in the welding process for two thicknesses of the top adherend, 2.37 mm and 3.55 mm.

### 3.2. Thickness of the Bottom Adherend

**Figure 8:**
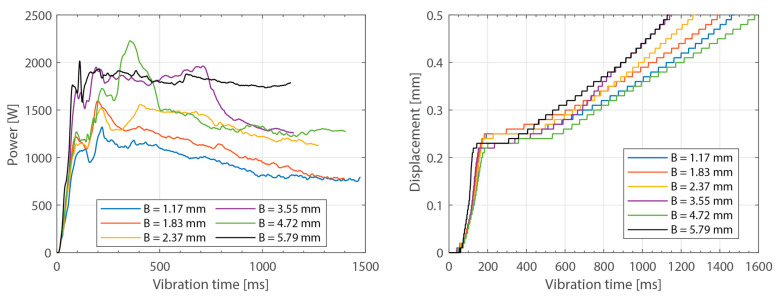
Consumed power (**left**) and downward displacement (**right**) curves for bottom adherend with varying thickness and discontinuous energy directors. One representative curve per thickness value. The welding parameters were 500 N force and 80 µm vibration amplitude. The top adherend’s thickness was 1.83 mm.

**Figure 9:**
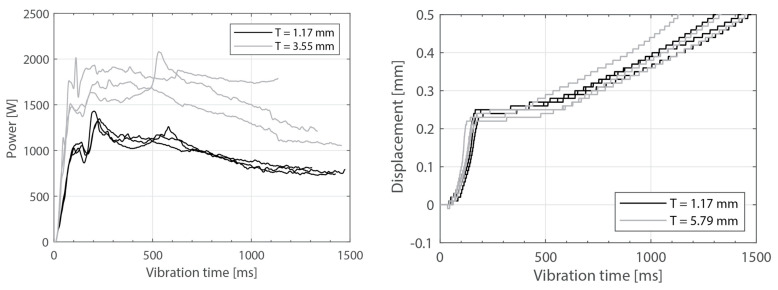
Consumed power (**left**) and downward displacement (**right**), three repetitions for two bottom adherend thickness values. The welding parameters were 500 N force and 80 µm vibration amplitude. The top adherend’s thickness was 1.83 mm.

**Figure 10:**
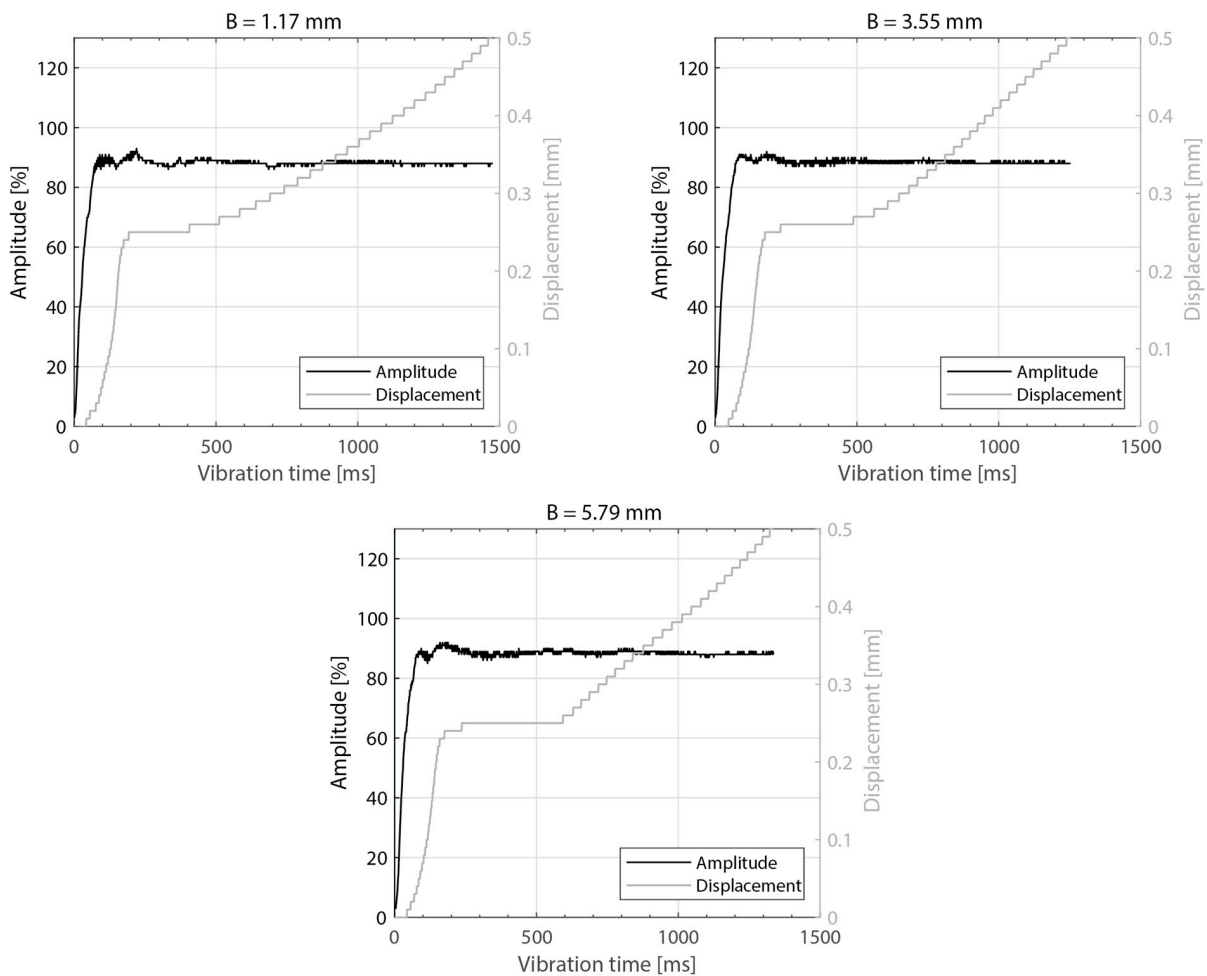
Amplitude and displacement curves for different thicknesses of the bottom adherend: 1.17 mm (**top-left**), 3.55 mm (**top-right**), and 5.79 mm (**bottom**). The welding parameters were 500 N force and 80 µm vibration amplitude. The top adherend’s thickness was 1.83 mm. The amplitude is provided as a percentage of the maximum amplitude the equipment can deliver.

**Figure 11:**
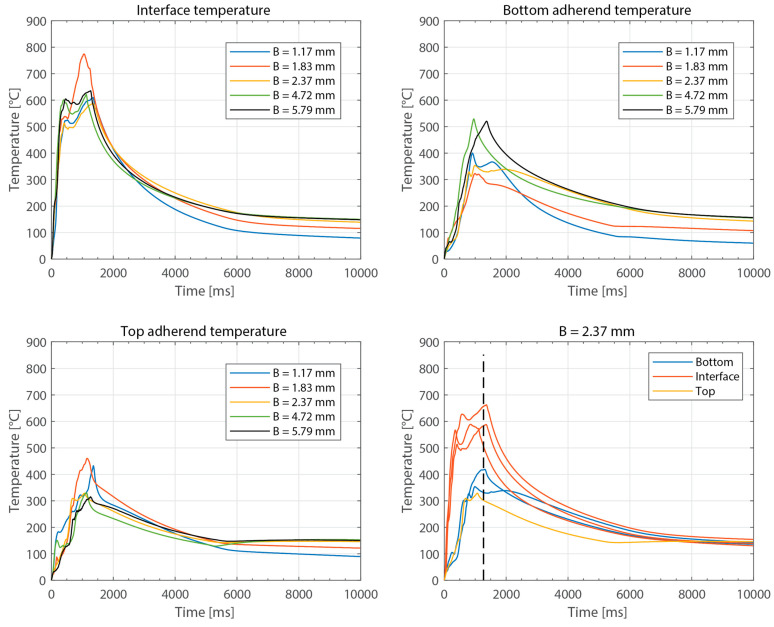
Representative temperature curves for different thicknesses of the bottom adherend: interface temperature (**top left**), bottom adherend temperature (**top right**), top adherend temperature (**bottom left**). Note that the temperature readings for 3.55 mm—thick bottom adherend are not present in these graphs since the decision to include this thickness in the study was made after the temperature measurements were performed. (**Bottom right**): three repetitions for a bottom adherend thickness of 2.37 mm. For the temperature in the top adherend, only one thermocouple survived, and, for the temperature in the bottom adherend, only two survived. The dashed line represents an estimate of the end of the vibrations. The welding parameters were 500 N force and 80 µm vibration amplitude. The top adherend’s thickness was 1.83 mm.

**Figure 12:**
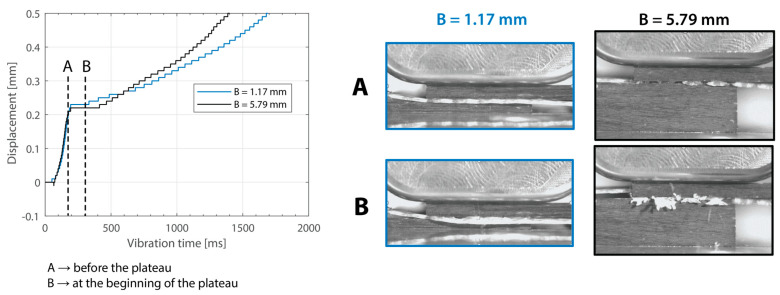
High-speed camera snapshots at different times in the welding process ((**A**) before the displacement plateau, (**B**) during the displacement plateau) for two thicknesses of the bottom adherend (1.17 mm and 5.79 mm). The welding parameters were 500 N force and 80 µm vibration amplitude. The top adherend’s thickness was 1.83 mm.

Figure 8, Figure 9, Figure 10, Figure 11 and Figure 12 show the same data as shown above but for the experiments in which the thickness of the bottom adherend was varied. In this case, it was possible to obtain results from the six thickness values originally planned, ranging from 1.17 mm to 5.79 mm. 

## 4. Discussion

This study aimed to gain an initial understanding of the effect of changing the thickness of the adherends during near-field ultrasonic welding of thermoplastic composites. The behavior of the process and the evolution of the welded joints when varying the thicknesses of the top and bottom adherends were studied using output data from the ultrasonic welder, temperature measurements, and high-speed camera recordings. 

As expected, increasing the thickness of the adherends increased the overall power consumption during the welding process. This effect was observed both when increasing the thickness of the top adherend (Figure 3) as well as when increasing the thickness of the bottom adherend (Figure 8). The main difference between the two cases was that increasing the thickness of the top adherend did cause a significant overshoot of the power peak observed at the onset of the process (Figure 3). This phenomenon may have been the reason why it was not possible to operate the ultrasonic welder for the highest top adherend thickness considered in this work, i.e., 5.79 mm. The observed differences may be related to the differences in wave transmission between the top and the bottom adherend. Indeed, every interface between two different media, i.e., sonotrode–top adherend, top adherend–energy director, and energy director–bottom adherend, will reflect a portion of the waves, resulting in a decrease in the vibrations transmitted across each interface. Consequently, it is sensible to assume that the process will be more sensitive to thickness changes in the top adherend since it receives the largest share of the vibration energy. It is, however, interesting to note that the thickness of the bottom adherend affects the power consumed throughout the process, even though the transmission of vibrations across the welding interface is said to significantly decrease once the energy director is molten [18]. 

**Figure 13:**
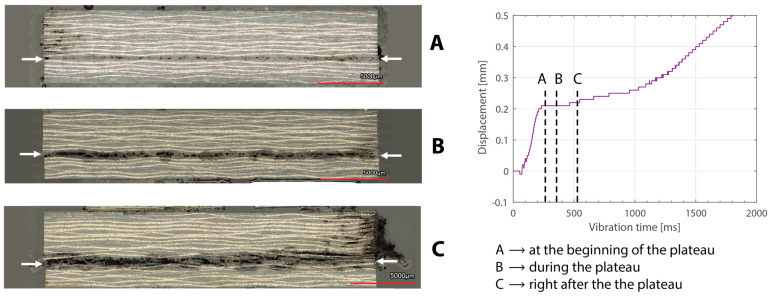
Cross-section micrographs of welded joints allowed to cool down without any consolidation pressure at different times in the welding process ((**A**) at the beginning of the displacement plateau, (**B**) during the displacement plateau, (**C**) right after the displacement plateau) for a top adherend thickness of 3.55 mm. The white arrows indicate the weldline. The welding parameters were 500 N force and 80 µm vibration amplitude. The bottom adherend’s thickness was 1.83 mm. Note that there is porosity observed at the weldline, which can be attributed to the absence of a consolidation phase. Note that the criteria for the micrographs were according to the position in the displacement curve, which is the reason why the times indicated in the graph do not match with the times provided in Table 2.

Beyond this, increasing the thickness of the top adherend was found to affect the downward displacement of the sonotrode during the welding process. As shown in Figure 1, the vertical displacement of the sonotrode is usually linked to the physical changes occurring at the welding interface during the welding process. In the case of a discontinuous energy director, as is the case in this study, the sonotrode will first travel downwards as it compresses the energy director until its original empty spaces are filled out with material [4]. At that point, the sonotrode will remain stationary (displacement plateau) until the energy director is molten and starts to flow, which will cause further downward displacement of the sonotrode (see Figure 1). All the curves in Figure 4 show roughly this behavior; however, it is interesting that, whereas for the 2.37 mm thick adherend and below, all the displacement curves overlap, for 3.55 mm and 4.72 mm thick adherends, the displacement curves show greater variability. One remarkable feature of the displacement curves corresponding to the thicker adherends is that the displacement plateau is reached at a lower downward displacement, which is surprising considering that both the energy director and the welding parameters are the same in all cases. The high-speed camera images in Figure 7 help to explain this behavior since they show that, in the case of the thicker top adherend, damage in the form of melting and fiber squeeze-out in the top adherend take place during the displacement plateau, as opposed to just melting of the energy director, as is the case for the thinner adherend. Temperature measurements (Figure 6) confirm that observation since they show a significant increase in the temperatures within the top adherend as its thickness increases. It should be noted that, due to potential errors in the readings caused by the interaction between the thermocouples and the high-frequency vibrations [19], the temperature data were only considered from a qualitative viewpoint. It is interesting to point out that, even though the images in Figure 7 do not clearly indicate melting of the energy director and hence the creation of a welded joint within the displacement plateau for the thicker adherend, the temperature readings at the welding interface seem to indicate the contrary since they are similar for the thin and the thick adherends (Figure 6). Cross-section micrographs of welds allowed to cool down without any consolidation pressure settle this argument by showing melting of the energy director (in the form of deconsolidation voids) within the plateau also in the cases of the thicker adherends (Figure 13). None of the effects described in this paragraph regarding heating of the adherends were, however, observed when increasing the thickness of the bottom adherend, as shown in Figure 9, Figure 11 and Figure 12. 

**Figure 14:**
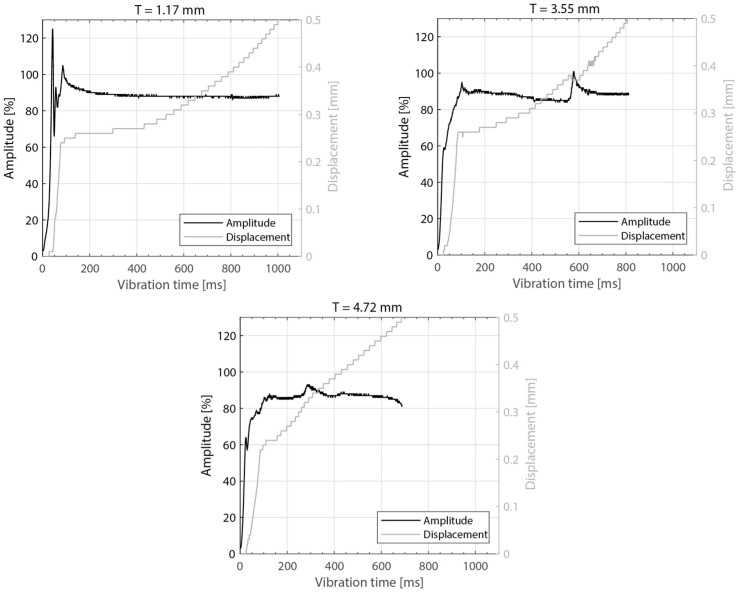
Amplitude and displacement curves for different thicknesses of the top adherend and increased welding force: 1.17 mm (**top-left**), 3.55 mm (**top-right**), and 4.72 mm (**bottom**). The welding parameters were 1500 N force and 80 µm vibration amplitude. The bottom adherend’s thickness was 1.83 mm. The amplitude is provided as a percentage of the maximum amplitude the equipment can deliver.

**Figure 15:**
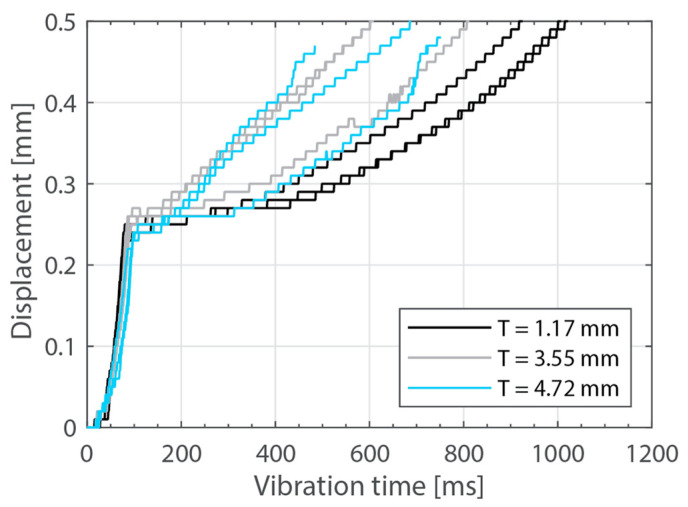
Displacement curves for increased welding force and different thicknesses of the top adherend (1.17 mm, 3.55 mm, and 4.72 mm). The welding parameters were 1500 N force and 80 µm vibration amplitude. The bottom adherend’s thickness was 1.83 mm.

**Figure 16:**
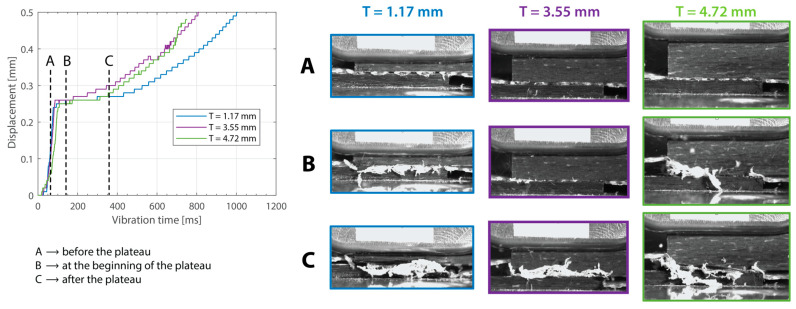
High-speed camera snapshots at different times in the welding process ((**A**) before the displacement plateau, (**B**) at the beginning of the displacement plateau, (**C**) after the displacement plateau) for three thicknesses of the top adherend (1.17 mm, 3.55 mm, and 4.72 mm). The welding parameters were 1500 N force and 80 µm vibration amplitude. The bottom adherend’s thickness was 1.83 mm.

**Figure 17:**
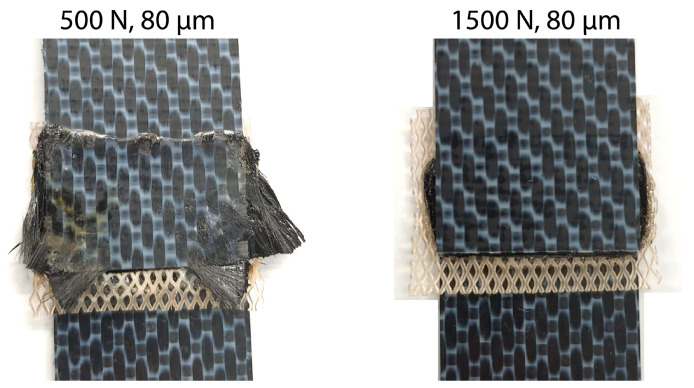
Comparison of the top adherend surface when welding with a force of 500 N (**left**) and 1500 N (**right**). This surface is in contact with the sonotrode during the vibration phase. The thickness of the top adherend was 3.55 mm, the thickness of the bottom adherend was 1.83 mm, and the welding amplitude was 80 µm. The joints were welded until a downward displacement of the sonotrode of 0.50 mm was reached.

The amplitude readings in Figure 5 and Figure 10 suggest the existence of significant amplitude oscillations, which were identified as hammering, in the case of the 3.55 mm thick top adherend. It should be noted that, of all the different thicknesses shown in those figures, that is the only case in which the displacement curve presents an “anomalous” behavior (Figure 3). Hammering is an unavoidable phenomenon in ultrasonic welding caused by the periodic loss of contact between the sonotrode and the top adherend [20]. To obtain an indication of whether hammering could be contributing to the temperature increase and melting observed within the thicker top adherends, an extra set of experiments with 1.17 mm, 3.55 mm, and 4.72 mm thick top adherends and a tripled welding force (1500 N) were carried out. It should be noted that increasing the welding force is known to improve the contact between the sonotrode and the top adhered [17], thereby reducing hammering [21], as also confirmed by the amplitude graphs in Figure 14 (see Figure 5 for reference). The new displacement curves (Figure 15) show, unlike those in Figure 4, overlapped displacement plateaus. Consistently, the high-speed camera images show no trace of melting and fiber squeeze-out within the adherends (Figure 16). Furthermore, a comparison of the surface of the top adherend, which is in contact with the sonotrode during the vibration phase, for both welding forces used in this study (Figure 17) shows the extent of the damage in the top adherend for the 500 N force case and the significant reduction in fiber squeeze-out and melting for the 1500 N case. Note that the observed fiber squeeze-out when welding with a 1500 N force is attributed to the fact that this corresponds to a full weld, which is known to be beyond the optimal point for joint strength in which fiber squeeze-out is expected to occur. These results are a clear indication of the role of hammering in the observed phenomena. Regarding the mechanism through which hammering might be contributing to the increased heating of the top adherend, the hypothesis is that it causes an oscillatory impact excitation, which, if close to the natural frequencies of the system, could result in an amplification of the cyclic strain and hence viscoelastic heating in the adherend. A precise modal analysis of the system as well as an analysis of the periodicity of hammering would be necessary to test such a hypothesis. Finally, the occurrence of more hammering in thicker adherends, which are nevertheless more compliant than thinner ones [19], can be explained by considering that, the more compliant a material is in static conditions, the more it behaves like a hard spring under dynamic loading [2].

## 5. Conclusions

The objective of this study was to provide an initial insight into the effect of the adherend thickness on the near-field static ultrasonic welding of fabric CF/LMPAEK thermoplastic composites. The results showed that increasing the thickness of the top adherend has a much more significant effect in the welding process than increasing the thickness of the bottom adherend. Indeed, increasing the thickness of the bottom adherend was only found to have an apparent effect on the overall consumed power. The increase in power caused by an increase in the thickness of the bottom adherend was, however, within the operating limits of the ultrasonic welder and did not cause any disruptions in the welding process (up to, at least, the maximum thickness considered in this study, i.e., 5.79 mm). Contrarily, increasing the top adherend thickness beyond 4.72 mm was found to preclude welding, likely due to the significant overshoot in the power required at the onset of the process. Furthermore, bulk heating in the top adherend during the welding process was found to increase as its thickness increased, likely as a result of increased hammering. Significantly increasing the welding force seemed to mitigate this issue while not having a negative effect on weldability. Further research on the implications of changing the thickness and process parameters on the joint strength is needed to gain more insight into some of the effects identified in this paper, e.g., increasing the welding force to mitigate negative effects when increasing the thickness of top adherend, and to determine processing windows for high-quality welds with thick adherends.

## Figures and Tables

**Table 1:** Thicknesses and laminate architectures used in this work.

Nominal Thickness (mm)	Laminate Architecture	Number of Plies
1.17	[(0/90)_2_]_s_	4
1.83	[(0/90)_3_]_s_	6
2.37	[(0/90)_4_]_s_	8
3.55	[(0/90)_6_]_s_	12
4.72	[(0/90)_8_]_s_	16
5.79	[(0/90)_10_]_s_	20

**Table 2:** Summary of the experiments presented in this study for the change in the top adherend’s thickness. The bottom adherend’s thickness was kept constant (1.83 mm). The experiments with thermocouples had all thermocouples present (interface, top adherend, and bottom adherend). T: top adherend’s thickness, TCs: thermocouples, d: displacement, t: time. Five repetitions (rep.) with TCs were completed for each case; however, only the repetitions in which the TCs survived are reported in the table.

T [mm]	Force [N]	Amplitude [µm]	Rep. without TCs	Rep. with TCs	Control	Purpose of the Experiments
1.17	500	80	3	1	d: 0.50 mm	To obtain power, displacement, amplitude, and temperature behavior during a full weld for comparison between the different thicknesses
1.83	500	80	3	1		
2.37	500	80	3	1		
3.55	500	80	3	2		
4.72	500	80	3	1		
3.55	500	80	3	0	t: 250 ms	To obtain micrographs at different positions of the displacement curve
3.55	500	80	3	0	t: 600 ms	
3.55	500	80	3	0	t: 800 ms	
1.17	1500	80	3	0	d: 0.50 mm	To study the effect of the thickness with a higher force value
3.55	1500	80	3	0		
4.72	1500	80	3	0		

**Table 3:** Summary of the experiments presented in this study for the change in the bottom adherend’s thickness. The top adherend’s thickness was kept constant (1.83 mm). The welding parameters were 500 N and 80 µm. The experiments with thermocouples had all thermocouples present (interface, top adherend, and bottom adherend). B: bottom adherend’s thickness, TCs: thermocouples, d: displacement, t: time. Five repetitions (rep.) with TCs were completed for each case; however, only the repetitions in which the TCs survived are reported in the table.

B [mm]	Rep. without TCs	Rep. with TCs	Control	Purpose of the Experiments
1.17	3	2	d: 0.50 mm	To obtain power, displacement, amplitude, and temperature behavior during a full weld for comparison between the different thicknesses
1.83	3	1		
2.37	3	3		
3.55	3	0		
4.72	3	2		
5.79	3	1		

## Data Availability

Data repository available at https://doi.org/10.4121/0de8b885-73bd-480d-86c8-138af34c0c6c.v1 (accessed on 26 October 2023).
